# Dysregulated Expression and Methylation Analysis Identified *TLX1NB* as a Novel Recurrence Marker in Low-Grade Gliomas

**DOI:** 10.1155/2020/5069204

**Published:** 2020-10-12

**Authors:** Hongzhou Duan, Zuozhen Yang, Chen Li, Jiayong Zhang, Shengli Shen, Changwei Yuan, Yingjin Wang

**Affiliations:** ^1^Department of Neurosurgery, Peking University First Hospital, Beijing 100034, China; ^2^MOE Laboratory of Biosystem Homeostasis and Protection, College of Life Sciences, Zhejiang University, Hangzhou, Zhejiang 310058, China; ^3^Department of Pediatric Cardiology, Beijing Anzhen Hospital, Capital Medical University, Beijing 100029, China

## Abstract

Low-grade gliomas (LGGs) are the most common CNS tumors, and the main therapy for LGGs is complete surgical resection, due to its curative effect. However, LGG recurrence occurs frequently. Biomarkers play a crucial role in evaluating the recurrence and prognosis of LGGs. Numerous studies have focused on LGG prognosis. However, the multiomics research investigating the roles played by gene methylation and expression in LGG recurrence remains limited. In this study, we integrated the TCGA and GEO datasets, analyzing RNA and methylation data for recurrence (R) and nonrecurrence (NR) groups. We found a low expression of *TLX1NB* and high methylation in recurrence patients. Low expression of *TLX1NB* is associated with poor survival (OS: *p* = 0.04). The expression of *TLX1NB* is likely to play a role in the prognosis of LGG. Therefore, *TLX1NB* may represent an alternative early biomarker for the recurrence of low-grade gliomas.

## 1. Introduction

Low-grade glioma (LGG) is an uncommon type of the primary central nervous system tumor classified by the WHO as Class I and II [1, 2]. Usually, LGG is inactive, and the main therapy strategy is complete surgical resection because this treatment can be curative. However, even if the tumor is resected, the tumor cells resistant to irradiation and chemotherapy may grow gradually; thus, the neoplasm can still relapse at some time point during the clinical course [3–6]. Due to recurrence and metastasis, the prognosis of LGG remains controversial [7, 8]. Because these tumors have a long asymptomatic natural history, whether patients with limited lesions and few symptoms are given active or delayed treatment and the timing of postoperative radiotherapy and chemotherapy have not been determined [9]. Thus, it is of great importance to understand the underlying molecular mechanisms governing LGG recurrence and to identify novel recurrence-associated biomarkers [5, 10].

Comprehensive multiomics provides a deeper and more comprehensive understanding of specific genes and enables us to identify functional genes for biomarker mining [11]. The Cancer Genome Atlas (TCGA) offers multidimensional maps of the key genomic changes in cancer, including alterations in DNA, RNA, copy number, and methylation [12].

Previous studies have revealed several biomarkers for LGG prognosis. For example, high methylation level of *HIST1H2BK* [13], the *MGMT* promoter [14], 1p/19q [15], the *TERT* promoter [16], *IDH* [16], and *mTORC1/2* [17] are associated with prognosis in LGG. However, the dysregulation of the expression of genes associated with LGG recurrence has not been fully characterized.

The objective of the present study was to explore possible clinical biomarkers of recurrence in LGG through multifaceted analysis of recurrence patterns in intracranial LGG.

## 2. Materials and Methods

### 2.1. Data Sources

RNA read count data, expression matrix data, copy number variation data, and DNA methylation data (Illumina Human Methylation 450 k Array) of LGG were obtained from The Cancer Genome Atlas (TCGA, https://cancergenome.nih.gov). Clinical information on LGG, including survival and recurrence data, was downloaded from the GDC Data Portal (https://portal.gdc.cancer.gov/) [12]. The gene expression matrix of GSE35158 was downloaded from the Gene Expression Omnibus (GEO) database.

### 2.2. Preprocessing and Analysis of RNA Data

We defined patients with recurrence as the case group and patients without recurrence as the control group. The RNA read count data were preprocessed via DESeq2 1.26.0 (R package) [18]; log2 fold change > 1 and adjusted *p* value < 0.05 were selected as the threshold. All differentially expressed genes were obtained. Bar plots of different genes were generated by ggplot2 3.3.0 (R package) [19]. Gene Ontology (GO), Kyoto Encyclopedia of Genes and Genomes (KEGG) enrichment analysis, and data visualization were performed by clusterProfiler 3.14.3 (R package) [20].

### 2.3. Preprocessing and Analysis of DNA Methylation Data

The DNA methylation data were preprocessed and normalized using minfi 1.32.0 (R package) [21]. Differentially methylated regions with a change value >0.1 or <-0.1 and a *p* value < 0.05 were selected as the methylation change threshold. All different methylated regions were obtained. Annotation was performed via the online tool wANNOVAR [22] (http://wannovar.wglab.org).

### 2.4. Overlap Statistics between RNA and Methylation and Copy Number Variation (CNV) Analysis

Upregulated RNA and hypomethylated genes or downregulated RNA and hypermethylated genes were extracted. Copy number variation analysis of target genes was performed by ggpubr 0.2.5 (R package) (https://github.com/kassambara/ggpubr).

### 2.5. Survival Analysis and Statistics

Survival plots of selected genes were generated by survival 3.1-12 (R package) and survminer 0.4.6 (R package) [23], and a *p* value < 0.05 was selected as the significance level.

### 2.6. GSEA Analysis

We calculated the correlation score between the target gene and all other genes and then ranked the correlation score from high to low. Finally, we obtained the gene list. We selected oncogenic gene sets (C6) as the input dataset, performed GSEA analysis by clusterProfiler 3.14.3 (R package) [20], and visualized pathways of interest by enrichplot 1.6.1 (R package) [20].

## 3. Results

### 3.1. Differentially Expressed Genes between Recurrence and Nonrecurrence Samples

**Figure 1:**
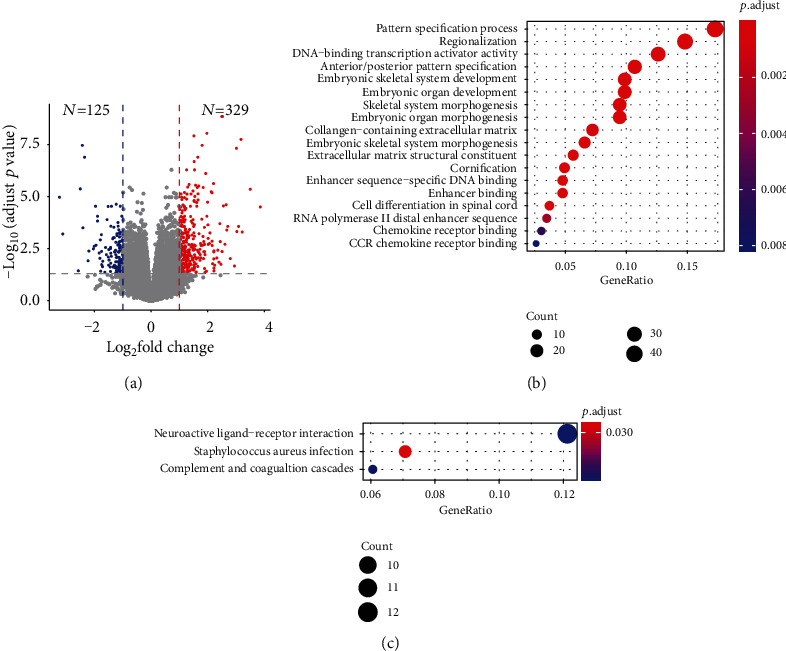
Differentially expressed genes between recurrence and nonrecurrence samples, number statics, and functional enrichment. (a) Upregulated (red point (*N* = 329)) and downregulated (blue point (*N* = 125)) genes between recurrence and nonrecurrence samples, adjusted *p* value < 0.05, fold change > 2 were selected as thresholds. (b) Differentially expressed gene-enriched GO terms; a *q* value < 0.05 was selected as the threshold. (c) Differentially expressed gene-enriched pathways; a *q* value < 0.05 was selected as the threshold.

We selected the samples with recurrence information and RNA sequencing data and eventually obtained 65 recurrence and 188 nonrecurrence data for further RNA expression analysis. We set log2 fold change > 1 and adjusted *p* value < 0.05 as a significant change threshold, and finally, we obtained 329 upregulated and 125 downregulated genes at the RNA level (Figure 1(a) and Table 1).

Through GO and KEGG functional analyses, the differentially expressed genes were determined to be primarily involved in neuroactive ligand−receptor interaction pathways (Figure 1(b)) and DNA-binding transcription activator activity (Figure 1(c)).

### 3.2. Identification of Methylation Regions between Recurrence and Nonrecurrence Samples

A total of 485,577 loci of DNA methylation were obtained from the TGCA database. After NA values were removed, 467,971 loci were retained for further analysis. Fifty-nine candidate DMRs were obtained from the analysis results. After annotation, removing unannotated DMRs, four hypermethylated regions were obtained for further cross-overlap analysis (Table 2).

### 3.3. Integrated Analysis for RNA Expression, DNA Methylation, and Copy Number Variation

**Figure 2:**
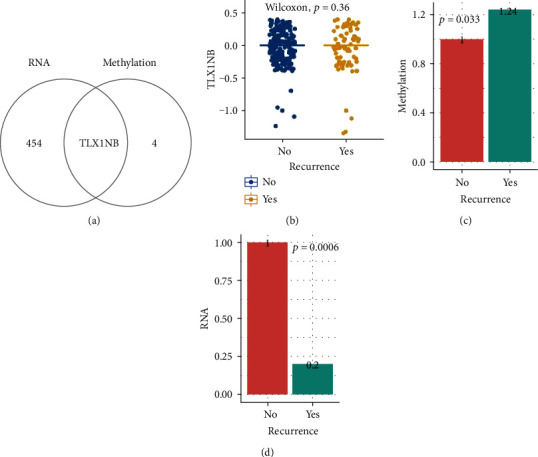
Gene overlap for RNA and methylation and copy number variation for *TLX1NB*. (a) Overlap for low expression at the RNA level (threshold: adjusted *p* value < 0.05, fold change > 2) and high methylation at the DNA level (threshold: methylation level change for DMRs (differentially methylated regions)) > 0.1). (b) Copy number variation analysis for *TLX1NB* between recurrence and nonrecurrence. A *p*-value < 0.05 was selected as the threshold. (c) Methylation level of *TLX1NB* between recurrence and nonrecurrence. A *p* value < 0.05 was selected as the significance threshold. (d) RNA expression level of *TLX1NB* between recurrence and nonrecurrence. A *p* value < 0.05 was selected as the significance threshold.

Genes were selected following the criterions: high methylation at DNA level and low expression at RNA level or low methylation at DNA level and high expression at RNA level. *TLX1NB* was identified by this approach (Figure 2(a)). The copy number variation of *TLX1NB* was investigated, and no difference between nonrecurrence and recurrence was found (Figure 2(b), *p* > 0.05). These results indicated that the downregulation of *TLX1NB* may not be associated with copy number alterations. To evaluate the methylation effect on *TLX1NB*, we compared methylation and RNA expression levels between recurrence and nonrecurrence groups. We found methylation of *TLX1NB* upregulated at 24% in the recurrence group compared with the control (Figure 2(c)), meanwhile at RNA expression level, the expression of *TLX1NB* downregulated at 80% (Figure 2(d)).

### 3.4. Gene Set Enrichment Analysis of TLX1NB

**Figure 3:**
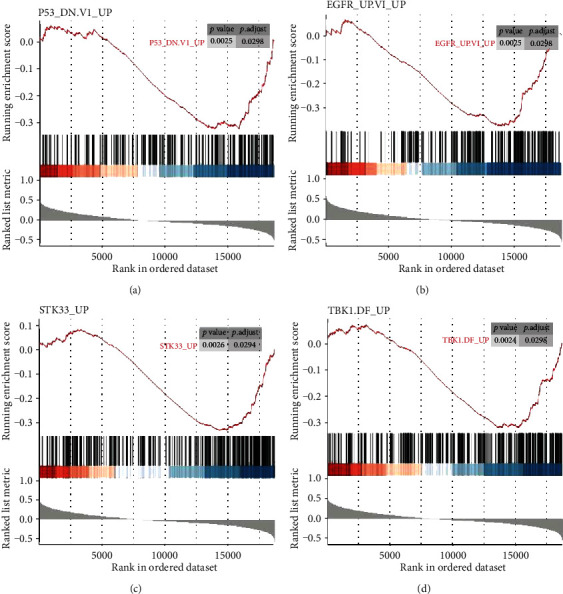
GSEA analysis of *TLX1NB* in the TCGA LGG dataset. Oncogenic gene sets (C6) were used as the input dataset, and a *p* value < 0.05 was selected as the significance threshold. *P53* (a), *EGFR* (b), *STK33* (c), and *TBK1* (d) gene sets were enriched.

To further investigate the potential functions of *TLX1NB*, GSEA was performed based on the LGG expression data. *TLX1NB* was negatively correlated with typical tumor driver genes, such as *P53*, *EGFR*, *TBK1*, and *STK33* (Figure 3). These results implied that *TLX1NB* is involved in typical cancer pathways and tumor proliferation processes.

**Figure 4:**
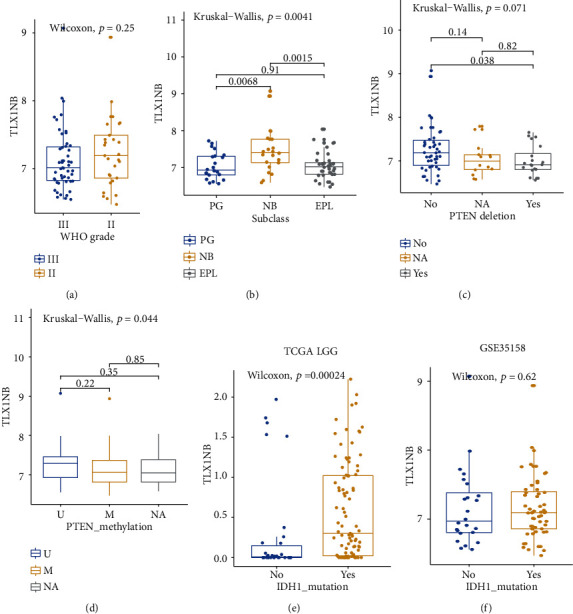
*TLX1NB* distribution in different subsets. (a) *TLX1NB* distribution between grade II and III LGG. A *p* value < 0.05 was selected as the significance threshold. (b) High expression of *TLX1NB* in the NB group compared with PG and EPL. A *p* value < 0.05 was selected as the significance threshold. NB: neuroblastic; PG: preglioblastoma; EPL: early progenitor-like. (c) Low expression of *TLX1NB* in the *PTEN* deletion group. A *p* value < 0.05 was selected as the significance threshold. (d) No difference for *TLX1NB* between *PTEN* methylation and unmethylation group. A *p* value < 0.05 was selected as the significance threshold. (e) High expression of *TLX1NB* in the *IDH1* mutation group in the TCGA dataset. A *p* value < 0.05 was selected as the significance threshold. (f) No difference for *TLX1NB* between *IDH1* mutation and wildtype group in GSE35158. A *p* value < 0.05 was selected as the significance threshold.

To understand the expression of *TLX1NB* in LGG patients in different subtypes, we employed dataset GSE35158 to analyze 80 patients' expression profiles. We observed that there was no difference in the *TLX1NB* expression between grade II and grade III patients (Figure 4(a), *p* = 0.25, Wilcoxon's test); however, there was a significantly increased *TLX1NB* expression level in the neuroblastic subtype, which was associated with Hu immunopositivity and a mature neuronal gene set in GSE35158 (Figure 4(b), *p* = 0.0041, Kruskal–Wallis test). We also found decreased expression of *TLX1NB* in the *PTEN* deletion group (Figure 4(c), *p* = 0.038, Kruskal–Wallis test). To explore the relationship between *TLX1NB*, *PTEN* promoter methylation, and *IDH* deletion, we analyzed *TLX1NB* expression and *PTEN* promoter in GSE35158 and *TLX1NB* expression and *IDH* mutation in TCGA and GSE35158. No significant change of *TLX1NB* expression was observed between *PTEN* methylation group and nonmethylation group in GSE35158 (Figure 4(d)). *TLX1NB* was significantly upregulated in the *IDH* mutation group in the TCGA dataset (Figure 4(e)); however, no difference was observed in the GSE35158 dataset (Figure 4(f)). The result for *IDH* mutation was controversial. So, we resume that *TLX1NB* has the potential to be a new biomarker in LGG associated with recurrence and prognosis.

### 3.5. TLX1NB Predicts Survival Level in LGG

**Figure 5:**
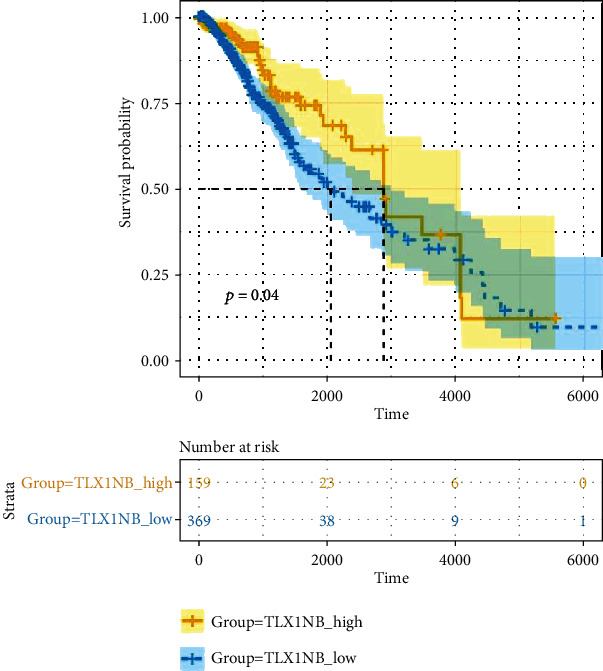
Overall survival plot for *TLX1NB* between the high and low groups in LGG. A *p* value < 0.05 was selected as the significance threshold.

From the previous results, we could assume that low expression of *TLX1NB* is associated with adverse consequences of LGG. Thus, we analyzed the prognosis of LGG patients in TCGA. Five hundred twenty-eight patients were divided into two groups: the high *TLX1NB* expression group and the low *TLX1NB* expression group. We observed that patients in the low *TLX1NB* expression group had shorter overall survival (OS) (Figure 5, *p* < 0.05, log-rank test).

## 4. Discussion

According to CBTRUS (Central Brain Tumor Registry of the United States), glioma is the most common CNS tumor, accounting for ~27% of CNS tumors [1] and leading to severe disability and mortality. According to the WHO central nervous tumor histological grading standards, such tumors such as grade I to II astrocytomas, papillary glioneuronal tumors, and vascular central gliomas are collectively referred to as low-grade gliomas [2]. LGGs account for 15% to 30% of all gliomas. LGGs are more common in children and young people with an average age of onset of 30 to 45 [4]. Although surgical resection is the main method of treatment, the tumor cannot be completely removed in the true sense due to the invasive growth of gliomas, the principle of tumor location, and the maximum protection of nerve function, which affects the survival of the patient [5]. Therefore, research that affects the risk of glioma recurrence may provide evidence to guide clinical treatment and facilitate the development of personalized strategies.

Regarding recurrence, previous studies revealed that for instance, high-level methylation of the *MGMT* promoter leads to hypermutation at recurrence, 1p/19q codeletion or *IDH* mutation is associated with longer overall survival and better treatment response, and *TP53* mutation is associated with a worse prognosis [5, 10, 14, 16, 24, 25]. However, the number of recurrence studies focusing on gene expression remains notably limited. In this study, we comprehensively analyzed TCGA and GEO data and observed that *TLX1NB* may be a potential recurrence biomarker. Previous studies have demonstrated that *TLX1NB* can be a prognostic lncRNA biomarker in lung adenocarcinoma [26]; however, there are no other studies revealing the role of *TLX1NB* in CNS cancer.

DNA copy number variations (CNVs) are an important component of genetic variation, affecting a greater fraction of the genome than single-nucleotide polymorphisms (SNPs) [27]. Therefore, we investigated the CNVs of *TLX1NB* and found no difference between the recurrence and nonrecurrence groups.

*TLX1NB* is adjacent to the *TLX1* gene, and we hypothesize that its role could be associated with *TLX1* function as a cis-regulatory factor [28]. Therefore, we correlated *TLX1NB* and *TLX1* and found that *TLX1NB* was positively coexpressed with *TLX1NB* (Supplementary Figure 1). *TLX1*, a member of the NK-linked or NK-like (*NKL*) subfamily, is involved in the specification of neuronal cell fates. Thus, we hypothesize that *TLX1NB* and *TLX1* together drive function. Our gene set enrichment analysis showed that *TLX1NB* was associated with typical cancer driver genes, such as *P53*, *EGFR*, *STK33*, and *TBK1*. These results demonstrate the role played by *TLX1NB* in cancer pathways. We also found that *TLX1NB* was negatively correlated with the transcription activating gene set (Supplementary Figure 2a) and the IL2 gene set (Supplementary Figure 2b).

## 5. Conclusion

We investigated the relationship between *TLX1NB* in LGG. *TLX1NB* is a predictor of LGG survival; reduced expression of *TLX1NB* worsens the prognosis and survival of LGG patients. *TLX1NB* probably affects LGG through the tumor activating pathway and could be a meaningful biomarker for LGG.

## Figures and Tables

**Table 1:** Differential expressed genes between the recurrence and nonrecurrence groups.

Numbers of genes	Log2 fold change cutofff	Adjusted *p* value
329	>1	<0.05
125	<-1	<0.05

**Table 2:** Differential methylated regions between the recurrence and nonrecurrence groups.

Chr	Gene.refGene	Value	Area	*p* value	fwer	*p*.valueArea	fwerArea
chr10	*GFRA1*	0.15761094	0.15761094	0.00147087	0.064	0.08985099	0.824
chr10	*GFRA1*	0.13806832	0.13806832	0.01701094	0.396	0.10360044	0.852
chr10	*TLX1NB*	0.11990024	0.23980049	0.01982478	0.444	0.03325446	0.568
chr3	*LINC02010*; *ZIC4*	0.13091445	0.13091445	0.04080067	0.656	0.12406472	0.892

## Data Availability

The raw data can download following the link in our manuscript, and analyzed data has been showed in table 1 and table 2. Regarding the intermediate process data we are willing to provide anytime when any researcher raise their request.

## Supplementary Materials

Supplementary Materials Supplementary Figure 1: correlation analysis between *TLX1NB* and *TLX1* (*R* > 0.8), and a *p* value < 0.05 was selected as the significance threshold. Supplementary Figure 2: GSEA analysis of *TLX1NB* in the TCGA LGG dataset. Oncogenic gene sets (C6) were used as the input dataset, and a *p* value < 0.05 was selected as the significance threshold. *ATF2* (Figures S2a) and *IL2* (Figures S2b) gene sets were enriched.
