# Supplementary material for: Dysregulated Expression and Methylation Analysis Identified TLX1NB as a Novel Recurrence Marker in Low-Grade Gliomas
Source: Int J Genomics. 2020 Oct 12;2020:5069204. doi: 10.1155/2020/5069204 (PMC7576335; doi:10.1155/2020/5069204)

**Supplementary material**

**Supplementary Figure legend**

Supplementary Figure 1. Correlation analysis between TLX1NB and TLX1, R>0.8, p value < 0.05 was selected as significant threshold.

Supplementary Figure 2. GSEA analysis of TLX1NB in TCGA LGG dataset. Oncogenic gene sets (C6) as input dataset, p value<0.05 was selected as significant threshold. ATF2 (Figures S2a) and IL2 (Figures S2b) gene sets were enriched.


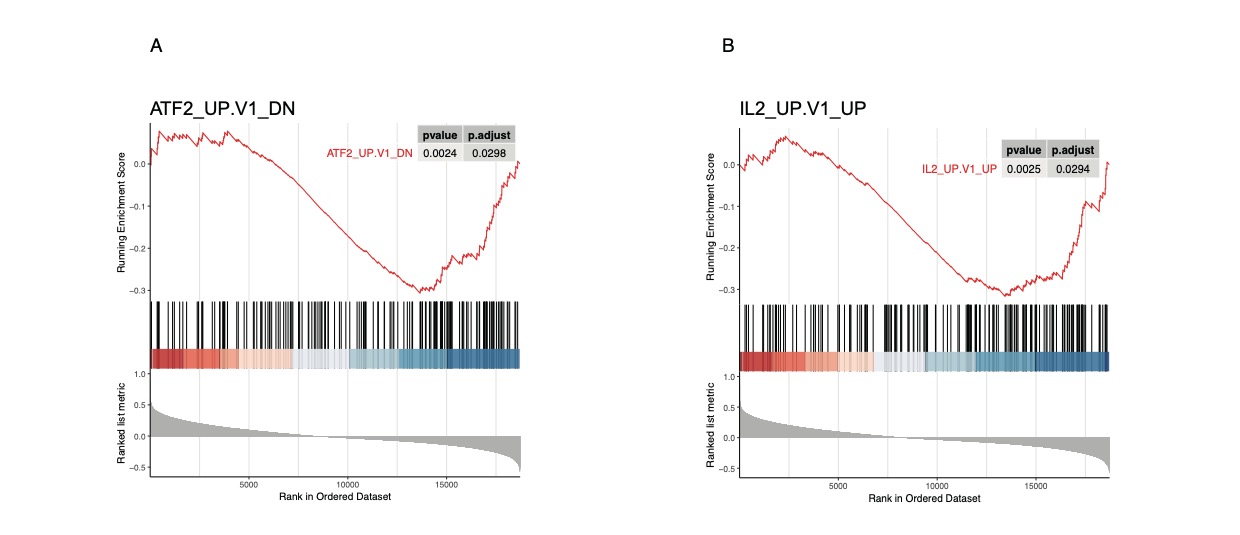

Supplement: Supplementary Materials — Supplementary Figure 1: correlation analysis between TLX1NB and TLX1 (R > 0.8), and a p value < 0.05 was selected as the significance threshold. Supplementary Figure 2: GSEA analysis of TLX1NB in the TCGA LGG dataset. Oncogenic gene sets (C6) were used as the input dataset, and a p value < 0.05 was selected as the significance threshold. ATF2 (Figures S2a) and IL2 (Figures S2b) gene sets were enriched. [file 5069204.f1.docx]
